# Concern and Helplessness: Citizens’ Assessments of Individual and Collective Action on the Provision of Environmental Public Goods in a Coastal City at Risk of Inundation

**DOI:** 10.1007/s00267-016-0730-2

**Published:** 2016-06-29

**Authors:** Sabrina Bunyan, Alan Collins, David Duffy

**Affiliations:** 1 Department of Accounting, Finance and Economics, Ulster University, Jordanstown Campus, Shore Road, Newtownabbey, Co. Antrim, BT37 0QB UK; 2 Economics and Finance, Portsmouth Business School, University of Portsmouth, Richmond Building, Portland Street, Portsmouth, PO1 3DE UK

**Keywords:** Climate change, Global public goods, Local public goods, Flood prevention, Coastal city

## Abstract

Survey data from a representative sample of 1005 households in the UK coastal city of Portsmouth are examined to discern commonalities and contrasts in their assessment of actions to address the related environmental threats of climate change and flooding. The city of Portsmouth is at risk of inundation from rising sea levels and the city has recent experience of flooding. A simple local and global public good framework is used to organize the understanding of reported attitudes and their determinants. The findings show that it is not always the same individuals who express concern about both climate change and flooding. Investigation into perceptions of helplessness in tackling climate change indicates that individuals more often perceived themselves to be helpless in tackling climate but perceived local collective action to be more effective. Individuals considered local collective action to be more effective in tackling climate change. Perceptions of individual helplessness are in turn related to reported concern. Several socioeconomic characteristics of individuals are shown to be useful in explaining the determinants of concern and perceptions of helplessness among respondents. As other cities face climate change-related challenges, the empirical findings, based upon attitudes from an alert urban population, are informative to policy design.

## Introduction

Climate change and its associated effects are a major issue of risk facing global populations. Increasingly, there are calls for action to tackle climate change through mitigation and adaptation efforts. To ensure that appropriate efforts are achieved in the UK, and elsewhere, an understanding of public perceptions of climate change is critical (Kellstedt et al. 2008; Whitmarsh 2009; IPCC 2014; Le Dang et al. 2014). Engaging citizens in climate change issues remains difficult. The rationale for this paper is to investigate the determinants and interrelationship between concern about climate change and flooding, as well as perceptions of helplessness with regard to tackling climate change, in a city at risk of inundation. A simple local and global public good framework is used to organize the understanding of reported attitudes and their determinants, at a city level, where experience of flooding and an understanding of the risks presented by climate change are prevalent.

The well-known collective action problem provides a standard explanation for expecting differences among private and public goods in terms of willingness to pay for them, or indeed willingness to expend individual effort in actually providing them. Uncertainty as to the value and likelihood of delivery of particular public goods can potentially further accentuate such differences. One such high-profile example of uncertainty is articulated in the form of climate change skepticism (Poortinga et al. 2011). This can range from straightforward existential challenges to the phenomenon, sliding down to varying degrees of acceptance of its presence, but qualified by atomistic feelings of helplessness, and thus doubts, as to the scope and value of prevention efforts. Attitudes to climate change mitigation (a global public environmental good) can be contrasted with those to more local (and potentially related) public environmental goods (such as flood prevention).

This paper reports on an analysis of survey data from residents in the city of Portsmouth, UK. Portsmouth is largely an island city under particular threat from coastal environmental degradation via flooding in its low-lying areas. This is likely to involve significant damage costs. Many Portsmouth residents have already endured episodes of flooding, most recently in 2000 and 2014. Given that it is a densely populated and relatively compact city, one might expect most residents will have read or heard news of these occurrences which could affect their attitudes to these concerns (Whitmarsh 2008). Local policy initiatives recognize the particular vulnerability of this city and view climate change as a key strategic risk (Portsmouth City Council 2012a). The key contribution of this paper is the analysis of novel survey data on the attitudes of city residents to the associated twin risk presented by flooding and climate change, as well as an analysis of their perceptions of helplessness in individual and local collective action in tackling climate change.

Other coastal settlements and urban centers must also be increasingly expecting to confront this situation in the UK and elsewhere (Yohe and Schlesinger 2002; Bronstert 2003; Næss et al. 2005; Hall et al. 2005, 2006). In economic terms, this problem actually comprises twin concerns with regard to risk relating to (1) a global public good (climate change mitigation) requiring global collective action to ensure provision, as well as (2) concerns about the need for flood prevention in Portsmouth, a related local public good requiring a geographically local collective solution. It is argued here that the local and global characteristics of these environmental public goods will condition attitudes among residents with respect to their concerns and perceptions of helplessness in the face of these environmental threats.

The empirical examination in this paper uses high-resolution city-specific attitudinal data, in a city at risk of inundation, with recent experience of flooding. It contributes to the literature by investigating the socioeconomic determinants of residents’ concerns regarding the associated environmental risks posed by climate change and flooding (O’Connor et al. 1999; Whitmarsh 2008; Kellstedt et al. 2008; Tjernstrom and Tietenberg 2008; van der Linden 2015). In addition, the same survey data investigate the socioeconomic determinants of residents’ perceptions of helplessness at the individual as well as the local collective action level in tackling climate change. Helplessness refers to an inability to influence or to produce a desired result. Personal helplessness, as such, corresponds to a lack of personal efficacy. There is a dearth of research on this matter. The interrelatedness of individual helplessness and concerns about flooding and climate change are also investigated (Kellstedt et al. 2008; Milfont 2012). The contribution of a civic activism variable, voluntary participation in local decision-making groups, in explaining concerns and perceptions of helplessness is also investigated. This variable has not typically been used in previous studies as far as the authors are aware. A novelty of this study is that it allows for a comparison of the socioeconomic determinants of concern about flooding and climate change as well as perceptions of individual and local collective helplessness in tackling climate change.

Empirical scrutiny of this attitudinal data is warranted to potentially inform policy design, guide appropriate technical communication to the public (Whitmarsh 2009), and also to provide some benchmark expectations about the nature of these public attitudes in relation to public environmental goods at different geographical scales in a city vulnerable to the effects of climate change. This is useful as “personal experience with noticeable and serious consequences of global warming is still rare in many regions of the world” (Weber 2006, p.103). Kellstedt et al. (2008) note that public risk perceptions drive policy as much as scientific risk assessments. As such, concern and perceived helplessness are key determinants in influencing policymakers and policy outcomes.

This paper is organized in the following manner. “Conceptual Framework and Related Literature” section outlines the conceptual framework and reviews the related literature. “The Portsmouth Context” section sets out the City of Portsmouth context and its environmental threats and policies. The research design and survey data are described in “Research Design: Survey-Based Evidence Regarding Attitudes to Climate Change and Flooding” section, while “Descriptive Statistics on the Interrelationships Between Attitudes” section provides some descriptive statistics on the interrelationships between attitudes to environmental issues. “Modeling Strategy” section sets out the modeling strategy, while “Results” section presents the results, “Discussion” section offers a discussion, and “Concluding Remarks” section provides some concluding remarks.

## Conceptual Framework and Related Literature

The conceptual framework used to organize the analysis of concerns about climate change and flooding, as well as perceptions of helplessness in tackling climate change, is a public goods approach. A public good, as defined by Samuelson (1954), is one “which all enjoy in common in the sense that each individual’s consumption of such a good leads to no subtraction from any other individuals consumption of that good” (p. 387). Additionally, public goods have the characteristic that once provided an individual cannot be excluded from the benefits of consuming that good. The characteristics and presence of public goods in the economy have been recognized in economics as a source of market failure (Black et al. 2012). The idea that private markets, and individual action, fail to provide public goods opens up an important role for the collective provision of these goods.

Climate change mitigation and flood prevention are two examples of public goods that fit the Samuelson definition [see Nicholson (1998, p. 744) on flood prevention and Sandler (2004) on climate change]. However, these are examples of public goods with differing characteristics (Kaul et al. 1999; Cullis and Jones 2009). The crucial distinction between climate change mitigation and flood prevention, in a public goods sense, arises due to the difference in the boundaries of nonexcludability in consumption. Flood prevention is a public good for which the benefits are nonexcludable; however, the extent of nonexcludability is limited by the spatial boundaries of a flood protection scheme. On the contrary, the benefits accrued through the mitigation of climate change are not specific to any area, but rather are enjoyed at the global level (Kaul et al. 1999; Sandler 2003).

The public goods framework emphasizes the need for collective action in the provision of climate change mitigation and flood prevention. It is possible to provide flood prevention through collective action at the local, regional, or national government level. However, a global public good, such as climate change mitigation, requires international cooperation to ensure effective provision, and as such, represents a “…collective action problem at the global scale” IPCC (2014, p. 5). Such global cooperation will involve substantial transaction costs and, unfortunately, “there is no past global collaboration of the scale required to achieve long lasting, broad-based” climate change mitigation (Libecap 2014, p. 462).

The collective action problem in the provision of climate change mitigation may give rise to perceptions of atomistic helplessness. The exacerbation of the collective action problem associated with climate change mitigation may elicit perceptions of helplessness among individuals regarding their own ability to tackle climate change as well as the ability of local government to do so. The novelty of the empirical analysis in this study is that it seeks to compare and contrast as well as explain differences in perceptions of helplessness in tackling climate change at the individual and the local collective levels. Understanding perceptions of helplessness is important as surveys have shown that perceptions of helplessness with regard to climate change have been identified as a reason for not taking action (Lorenzoni et al. 2007).

The collective action problem associated with the provision of the public goods, flood prevention, and climate change mitigation would be expected to elicit differences in concerns among individuals. Although, provision mechanisms for global and local public goods are different, concerns about the risks presented by flooding and climate change are interrelated. Flooding risk is a subset of the consequences of climate change. This association is recognized by the public who perceive flooding to be one of the impacts of climate change, see for example Upham et al. (2009).

In general, public awareness and concern about climate change is high (Upham et al. 2009). Despite this, climate change is often misunderstood by the general public, who, among other things, inappropriately attribute weather events to climate change (Kempton et al. 1996). Recent studies have continued to find that the general public hold incorrect beliefs about climate change and its anthropogenic causes (Reynolds et al. 2010). Moreover, despite growing scientific consensus on the existence of climate change and its anthropogenic causes, recent studies, since the latter part of the 2000s, have found that public attitudes to climate change have become more skeptical and polarized, see Capstick et al. (2015a, b) for a review. Despite this growing climate change skepticism, clear majorities in many countries still recognize and are concerned about climate change.

However, levels of concern expressed by the general public are not to the same degree as those expressed by climate change scientists (Weber 2010). For the general public, other economic and social concerns are often found to be more pressing (Bord et al. 1998; Eurobarometer 2008). Moreover, concern about climate change does tend to fluctuate with weather events and media attention (Bord et al. 2000). The 2007 data used in this study need to be understood in this context.

An explanation as to why other concerns may be more pressing is provided by construal level theory (Liberman and Trope 2008). This theory emphasizes how psychological distance may hinder an individual’s ability to engage with distant threats.^1^ Evidence finds that climate change risk is one such psychologically distant risk for many people (Lorenzoni and Pidgeon 2006; Spence et al. 2011, 2012). Besides other more pressing concerns, the psychological distance of climate change is increased as (a) the impacts of climate change are often viewed to be a concern for future generations and other regions, (b) others,’ for example industry, actions are viewed as the primary causes of climate change, (c) the responsibility for tackling climate change is assigned principally to government, and (d) there is a high degree of uncertainty surrounding climate change (Upham et al. 2009; Poortinga et al. 2011).

Public perceptions of climate change are in part influenced by a lack of personal experience with the impacts of climate change (Weber 2010). Psychological distance can be reduced through personal experience. Indeed, Spence et al. (2011) found that those with the experience of flooding expressed more concern about climate change. Moreover, Le Dang et al. (2014) found that experience of some climate change phenomena is associated with greater perceived risks from climate change. However, Whitmarsh (2008) found that flood victims differ slightly from the rest of the population in their understanding of and responses to climate change. Experience of flooding, a known consequence of climate change, in a low-lying coastal region would be expected to reduce the psychological distance of both climate change and flooding in the Portsmouth context. This paper provides an understanding of the intertwined nature of concerns about flooding and climate change, as well as their socioeconomic determinants, which is useful in this context.

Previous studies have found that attitudes toward climate change are determined by a range of demographic variables. Kellstedt et al. (2008) and Tjernstrom and Tietenberg (2008) found that younger people are more likely to be concerned about climate change. Additionally, the role of available information is also found to play a role in determining attitudes through both general knowledge and subject-specific scientific knowledge of climate change. Tjernstrom and Tietenberg (2008) found that general knowledge, measured by a higher level of education, is associated with concern about climate change. Other studies found that specific knowledge of climate change science is associated with increased levels of concern (Wu and Cutter 2011; Milfont 2012). On the contrary, Whitmarsh (2011) found a little relationship between knowledge, education, and climate change concern, while O’Connor et al. (1999) found that education resulted in less concern. Income levels have also been found to be associated with lower levels of climate change concern (O’Connor et al. 1999; Tjernstrom and Tietenberg 2008). Urban residents have been found more likely to be concerned about climate change (Tjernstrom and Tietenberg 2008). With respect to flood risk, urbanization can increase risk by reducing the permeability of ground surfaces (Parker 1999). Studies on risk perception have documented that women perceive the world as more risky than men (O’Connor et al. 1999; Kellstedt et al. 2008); more specifically, van der Linden (2015) found females to be more concerned about climate change.

Perceptions of helplessness might be expected to be greater among those who have experienced flooding, or those who live in flood risk regions, that could be attributed to climate change. This would arise out of an individual’s inability to take meaningful action to tackle climate change. On the contrary, in accordance with goal-setting theory, Spence et al. (2011) suggests that “if people are better able to relate to the potential consequences of climate change impacts, they may also be more likely to feel that their behaviour can lead to changes in these impacts” [p. 2]. Indeed, Spence et al. (2011) show that those who experience the effects of climate change feel more confident that their actions will have an effect. Milfont (2012) found a relationship between concern and helplessness, with greater levels of concern leading to lower levels of helplessness. There is a dearth of studies examining the determinants of helplessness with regard to tackling climate change, either through mitigating or adaption efforts.

## The Portsmouth Context

There is a growing awareness in society of the risks presented by climate change and an associated concern with regard to these risks (Eurobarometer 2008). In recent years, information on the causes and consequences of climate change has increasingly entered the public awareness. In the UK, it has been recognized that wetter winters and rising sea levels are associated with climate change and one of its biggest impacts is flooding (Environmental Agency 2005; Pall et al. 2011). Climate change has also been identified as a driver of sea-level change (Solomon et al. 2007). Major changes in sea level are expected to impact upon vulnerable populations in low-lying coastal areas such as the City of Portsmouth (Parry et al. 2007). The risk of flooding in Portsmouth is very high with around one-third of the City’s land area, and 20 % of the city’s dwellings, on a tidal floodplain (Portsmouth City Council 2012b). This figure will only increase with a rise in sea levels which are predicted to be between 0.7 and 1.9 m over the next 70 years (Portsmouth City Council 2012b).

Individuals who live in low-lying coastal areas, such as Portsmouth, have been shown to have an increased awareness of the potential negative impacts of climate change (Brody et al. 2008). In recent years, Portsmouth experienced flooding in 2000 and in 2014. There were also several previous experiences of flooding in the City. The experience of excessive rainfall in England would appear to be becoming increasingly regular with the winter of 2013/2014 recorded as the “…wettest on record” (Department for Communities and Local Government 2014). It is difficult scientifically to attribute specific weather events to climate change; however, survey evidence reported that a majority of the British public associated the 2014 winter floods with climate change (Capstick et al. 2015a, b). Given that Portsmouth is a densely populated and relatively compact city, one might expect most residents will have read or heard news of these occurrences which could affect their attitudes to these concerns (Whitmarsh 2008). Many parts of the UK experienced large-scale flooding during 2007 making flooding a key media and political concern at the time. This experience in Portsmouth of flooding and climate change is not typical as “personal experience with noticeable and serious consequences of global warming is still rare in many regions of the world” (Weber 2006, p. 103).

Collective efforts to tackle climate change in Portsmouth have been undertaken to both mitigate and adapt to the effects of climate change. The Department for Environment Food and Rural Affairs has responsibility for flood and coastal defense in England, although delivery, in Portsmouth, is the responsibility of Portsmouth City Council and the Environment Agency (Portsmouth City Council 2008). A key policy objective, of Portsmouth City Council, is to protect Portsmouth against the impacts of climate change with regard to flood risk from the sea. Abandonment of the areas at risk to flooding is thought to be an unrealistic option (Portsmouth City Council 2012b). Flood risk minimization also informs the City Council’s planning regulations. Increased flood risk will be controlled through the maintenance and improvement of the city’s flood defenses.

In comparison to adapting to increased flood risk, there is less local collective action can achieve in mitigating global climate change. Despite this, Portsmouth City Council view themselves as having a key role in promoting environmentally sustainable development, environmentally friendly means of travel, and the general promotion of a low-carbon city. The City Council’s “Building resilience to climate change” policy sets out to ensure that the city is resilient to climate change and sets out actions to be taken where appropriate (Portsmouth City Council 2012a). The policy recognizes the City’s vulnerability and identifies climate change as a strategic risk. The need for major projects such as costal defenses and current infrastructural improvements are recognized.

The availability of high-resolution data from a city level is useful in informing policy development beyond the Portsmouth context. The Portsmouth context provides an insight into attitudes related to climate change in a city, in a developed economy, which is at the sharp end of experiences of climate change. This is a community with a great deal of experience of the vulnerabilities associated with flooding and, consequently, is a highly alert public to these issues. Other regions increasingly face such risks as the climate changes. Understanding the attitudes of Portsmouth residents provides, as such, an early insight that can help advance policy design.

## Research Design: Survey-Based Evidence Regarding Attitudes to Climate Change and Flooding

### Survey Description

Survey data were obtained from a survey of 1094 households, commissioned by Portsmouth City Council, and conducted by Ipsos Mori. Of the sampled households, 1005 households responded to the survey giving a 92 % response rate. Respondents were surveyed face-to-face in their own homes between October 6 and December 14, 2007. Stratified sampling methods, based on the 2001 census, were used to randomly select respondents from sampling points across the city.^2^ Responses were only obtained from those over the age of 16.

In addition to detailed demographic information, the survey also consisted of 54 questions on a wide range of topics, such as digital accessibility, fear of crime, and a range of other quality-of-life issues and local city priorities. The majority of questions are related to existing measures used in previous surveys, while others were one-off questions to capture public opinion on current topics of interest.

### Dependent Variables

The survey consisted of four questions relating to environmental public goods.^3^ These included a question each on concern about climate change and concern about flooding affecting Portsmouth, and also a question each on perceived helplessness in tackling climate change at an individual and at a local government level. These questions provided the data for the dependent variables in this study. The dependent variables were measured in the form of a five-point Likert scale (Lewis-Beck et al. 2003). Details of the questions and responses used as dependent variables in this study are provided in Table 1 below.

**Table 1:** Responses to questions on concerns about climate change and flooding as well perceptions of helplessness at the individual and local collective levels

Questions	Frequency	Percentage (*N* = 1005)
How concerned are you about climate change affecting Portsmouth?		
1 = a great deal	225	22.4
2 = a fair amount	383	38.1
3 = not very much	261	26.0
4 = not at all	116	11.5
How concerned are you about the possibility of flooding affecting Portsmouth?		
1 = very concerned	186	18.5
2 = fairly concerned	383	38.1
3 = not very concerned	311	30.9
4 = not at all concerned	108	10.7
How much influence do you think personally can have on tackling climate change in Portsmouth?		
1 = none at all	143	14.2
2 = not very much	375	37.3
3 = a fair amount	389	38.7
4 = a great deal	76	7.6
How much influence do you think the council can have on tackling climate change in Portsmouth?		
1 = none at all	55	5.5
2 = not very much	231	23.0
3 = a fair amount	406	40.4
4 = a great deal	284	28.3

Portsmouth Residents’ Survey, 2007. Missing responses are not shown in the table

Attitudinal questions, such as the questions here on helplessness and concern, have the limitation that they were open to interpretation by respondents. Moreover, this study is constrained by the use of secondary data. Respondents were asked about perceptions of helplessness in “tackling” climate change. Potentially, tackling could have been understood as referring to both, or either, adaptation (building flood defenses) and/or mitigation. Additionally, the City Council’s policy response to climate change is both to adapt (building/maintaining flood defenses) and to contribute to mitigation efforts, though, for example, the general promotion of a low-carbon city. As such, the term tackling captures the idea among respondents that they as individuals, or through local collective action at the city level, can do something to deal with the problem of climate change. Hence, helplessness is understood to capture a lack of influence in tackling climate change, where tackling refers technically to a combination of both adaptation and mitigation efforts. At the local collective level, city council helplessness is assumed to refer to helplessness at the local collective level in absolute terms rather than relative to another medium-size coastal city in the UK or Europe.

Table 1 shows that reported concern about both environmental public goods is similar. If concern is those respondents who stated some level of concern, those in response categories 1 and 2, a total of 569 (56.6 %) respondents express concern about flooding, while a total of 608 (60.5 %) respondents express concern about climate change. If helplessness is those who stated some level of helplessness, again those in categories 1 and 2, a total of 518 (51.5 %) respondents reported perceptions of individual helplessness in preventing climate change, while substantially fewer respondents reported helplessness at the local collective level, 286 (28.5 %) respondents. Reported helplessness in mitigating climate change is much greater at the individual than at the local collective level.

### Independent Variables

A range of demographic and other independent variables, as suggested by the literature reviewed, are included to explain the environmental attitudes of respondents. The formal modeling section of this paper uses the independent variables to disentangle the determinants of the differences in attitudes of concern and perceptions of helplessness. These independent variables are detailed in Table 2 below.

**Table 2:** List of explanatory variables and definitions

Explanatory variables	Frequency	Percentage (*N* = 1005)
Age		
16–24	166	16.5
25–34	158	15.7
35–44	213	21.2
45–54	132	13.1
55–64	126	12.5
65–74	107	10.6
75+	103	10.2
Income		
Under £199 per week	302	30.0
£200–399 per week	305	30.3
£400–599 per week	178	17.7
£600+ per week	220	21.9
Education^a^		
Third level	182	18.1
Second level	431	42.9
Neither second nor third level	273	27.2
Housing tenure		
Owned	646	64.3
Rent	336	33.4
Gender		
Male	473	47.1
Female	532	52.9
Presence of young people in household		
Young people present	400	39.8
No young people present	605	60.2
Disability		
Disability	154	15.3
No disability	851	84.7
Home contents insurance		
Home contents insurance	777	77.3
No home contents insurance	187	18.6
City center dweller (location)		
City center	129	12.6
Outside city center	858	85.4
Voluntary participation in decision-making groups^b^		
Participates	104	10
Does not participate	901	90

Portsmouth Residents’ Survey, 2007. Missing responses are not shown in the table

^a^This is a measure of years of schooling. Second level corresponds to high school, A Level, GCSEs or equivalent, while third level refers to a university degree or equivalent

^b^This variable has been included as a proxy to capture prosocial behavior

Age is a measure of the age in years of the respondents. Age was grouped in ten-year bands and ranged from 16 to 95 years. Previous research found that age influenced attitudes to climate change (Kellstedt et al. 2008; Tjernstrom and Tietenberg 2008). The income variable measures total household gross income. Respondents were asked to indicate the group in which they would place their and their partner/spouses’ current total gross income from all sources before deductions of tax and national insurance—that is income from work and any other sources, such as pensions and benefits. Previous research found income to influence attitudes to climate change (O’Connor et al. 1999; Tjernstrom and Tietenberg 2008).

Education is a measure of the respondent’s academic qualifications. The data distinguish between three categories of respondents, those with at least a third-level education (degree of equivalent), second-level education (high school, A Level, GCSEs or equivalent), and those with neither second- nor third-level education. Education had previously been found to influence attitudes to climate change (O’Connor et al. 1999; Tjernstrom and Tietenberg 2008).

Gender is a measure of the sex of respondents. Studies on risk perception have documented that females perceive the world as more risky than men (O’Connor et al. 1999; Kellstedt et al. 2008; van der Linden 2015). Housing Tenure is a measure of a respondent’s housing status. This variable distinguishes between respondents who own their property and those who rent (from the council, housing association, or private landlord). The rationale for including housing tenure is due to the a priori expectation that home owners’ attitudes to climate change may differ to renters in a city at risk of inundation. Home contents insurance measures whether or not the household has home contents insurance. The rationale is that there may be a relationship between concern and the choice of taking up home contents insurance.

The presence of young people in the household measures whether any dependent children under the age of 18 live in the household. This variable is included to take account of the potential impact of personal issues, such as supporting a family, which may be more pressing and less psychologically distant than climate change and flood threats (Upham et al. 2009). Disability measures whether the respondent has any long-term illness, health problem, or disability which limits their daily activities or the work they can do. Again, as with the presence of young people in the household variable, this variable is included to take account of other more pressing concerns the respondent may have.

The city dweller variable captures whether the respondent resides in the city center or not. The city center of Portsmouth is highly urbanized and contains some of the most deprived neighborhoods in Portsmouth (Bunyan and Collins 2013). This variable has been included as previous research found that urban residents are more likely to be concerned about climate change (Tjernstrom and Tietenberg 2008). Additionally, with respect to flood risk, urbanization can increase the risk by reducing the permeability of ground surfaces (Parker 1999).

Voluntary participation in decision-making groups captures participation in local decision-making groups covering a range of issues and services across the city. This includes participation in decision-making-groups affecting education, health, crime, race, housing, urban regeneration, old age services, youth services, or any other decision-making groups. As such, this variable captures voluntary civic activism in the community as well as a willingness to participate in effecting change. The a priori expectation is that this variable will impact upon attitudes to climate change, as it indicates heightened concern for community issues, including environmental issues.

## Descriptive Statistics on the Interrelationships Between Attitudes

This section provides an initial examination of some descriptive statistics on the interrelatedness of concerns about flooding and climate change and perceptions of helplessness at the individual and local collective levels. With regard to concern, 97 % of respondents answered both questions related to concern about flooding and climate change. Details on the cross tabulations of reported concern with both flooding and climate change are given in Table 3.

**Table 3:** Individual concern about flooding and climate change

	Concerned with climate change (*N* = 608) (%)	Not concerned with climate change (*N* = 377) (%)
Concerned with flooding (*N* = 569)	69.7	35.5
Not concerned with flooding (*N* = 419)	29.3	62.3
No answer (*N* = 17)	1	2.2
Total (*N* = 1005)	100	100

The cross tabulation of the two variables reveals that it is not always the same respondents who are concerned about the two environmental threats. Of the 608 respondents who reported concern about climate change, 29.3 % are not concerned about flooding. Also, of the 377 respondents who reported not to be concerned about climate change, 35.5 % reported to be concerned about flooding. Understanding the attributes of individuals who expressed these differences in concern is informative and a contribution of the formal modeling section of this paper.

Turning next to perceptions of helplessness, Table 4 provides a cross tabulation of perceptions of helplessness in tackling climate change at the individual and local collective action levels. Of the 286 respondents who reported helplessness at the local collective level, 88.1 % also perceived themselves as individuals to be helpless. However, of the 690 respondents who perceived the City Council to be effective in tackling climate change, 36.1 % perceived themselves as individuals to be helpless. The formal modeling section disentangles the determinants of helplessness at the individual and city council levels.

**Table 4:** Personal and collective helplessness in tackling climate change

	Local council collective influence (*N* = 690) (%)	Local council collective helplessness (*N* = 286) (%)
Individual influence (*N* = 465)	62.8	10.1
Individual helplessness (*N* = 518)	36.1	88.1
No answer (*N* = 22)	1.1	1.8
Total (*N* = 1005)	100	100

## Modeling Strategy

The formal modeling strategy in this paper is to estimate four separate logistic models to explain the following attitudes: (1) concern about flooding, (2) concern about climate change, (3) personal helplessness in tackling climate change, and (4) City Council (local collective) helplessness in tackling climate change. The explanatory variables in all models include the following: gender, age, household income, education level, disability, city center dweller, young people in household, housing tenure, and voluntary participation in local decision-making groups. The first two models include the variable home contents insurance, and the rationale is to capture an expected relationship between concern and the choice of taking up home contents insurance. Due to the large number of categorical variables, a stepwise approach is taken in order to run a more parsimonious model. This stepwise approach involved removing the most statistically insignificant variables. For robustness purposes, and to further examine the determinants of any interrelatedness and differences in concerns regarding climate change and flooding, a multinomial regression was also estimated to take account of the joint determination of concern about climate change and flooding.^4^

In an attempt to help elucidate on the relationship between concern and perceived helplessness, individual helplessness is included as an explanatory variable in the models explaining concern as in Kellstedt et al. (2008). A relationship is expected between these variables as self-efficacy may influence concern (as well as vice versa) through mechanisms of cognitive dissonance and identity threat (e.g., if you feel you cannot act to reduce a risk, this can lead to denial in order to prevent anxiety).

There are several approaches available to modeling attitudinal dependent variables, which are measured on a Likert scale, see Lewis-Beck et al. (2003) for a review. Given the ordered nature of the data, initially, an ordered logistic model was estimated. A key assumption of the ordered logistic model is that of proportional odds or parallel regression lines. The estimated ordered regression models were not found to be robust to this assumption. Hence, the choice is then to run either a multinomial logistic regression model, or to collapse the dependent variables into two categories. Collapsing the categories is considered the most feasible option when there are few categories and when the categories can be legitimately collapsed (Lewis-Beck et al. 2003). The focus in this study is on two categories of individuals in each model: (1) those that are concerned, or not concerned about climate change, (2) those that are concerned, or not concerned about flooding, (3) those perceiving individual helplessness, or not, and (4) those perceiving local collective helplessness, or not.^5^ This approach of collapsing attitudinal data was used in previous studies, for example, see Whitmarsh (2008) and Dustmann and Preston (2001).^6^

As is the case with many surveys of this nature, approximately one-third of the sample did not reveal their gross household income. To enable the use of these observations, an income variable was imputed using multiple imputations.^7^

## Results

The results of the logistic regressions explaining concern about flooding and climate change are shown in Table 5, while the results of the logistic regressions in explaining perceptions of individual and local collective helplessness are provided in Table 6. For each explanatory variable, the coefficient value, the standard error, the *p* value, as well as the odds ratio (Exp(*β*)) are presented. The pseudo *R*-square results of Cox & Snell and Nagelkerke are used to assess the overall goodness of fit of the models. The classification rate is also presented. The results are robust to tests of multicollinearity.^8^

**Table 5:** Logistic regression results explaining residents’ concern about climate change and flooding

Variables	Model 1: concerned about flooding				Model 2: concerned about climate change			
	*β*	S.E.	*p* value	Exp(*β*)	*β*	S.E.	*p* value	Exp(*β*)
Gender^a^	0.354	0.148	0.017	1.424	0.399	0.157	0.011	1.491
Income^b^			0.127				0.120	
<£199	0.468	0.242	0.053	1.597	0.379	0.259	0.143	1.461
£200–399	0.519	0.225	0.021	1.681	−0.090	0.235	0.703	0.914
£400–599	0.322	0.234	0.168	1.380	0.218	0.252	0.388	1.243
Education^c^			0.002				0.007	
Second level	−0.710	0.206	0.001	0.492	−0.662	0.220	0.003	0.516
Neither second nor third level	−0.706	0.242	0.004	0.494	−0.701	0.255	0.006	0.496
Home contents insurance^d^	0.599	0.201	0.003	1.820	0.562	0.211	0.008	1.754
Individual helplessness^e^	−0.513	0.147	0.001	0.599	−1.288	0.159	0.000	0.276
Voluntary participation in local decision-making groups^f^	0.498	0.244	0.041	1.646	0.637	0.266	0.017	1.890
Constant	0.045	0.284	0.874	1.046	0.880	0.304	0.004	2.410
*R* ^2^ Cox–Snell	0.059				0.131			
*R* ^2^ Nagelkerke	0.079				0.178			
Hosmer and Lemeshow statistic	3.805				9.839			
*p* value	0.874				0.276			
Classification (%)	60.4				68.2			
*N*	820				823			

Reference categories used in the model:

^a^Males

^b^Income group £600+ per week

^c^Third level

^d^No home contents insurance

^e^No disability, not helpless

^f^Does not participate

**Table 6:** Logistic regression results explaining perceptions of individual and local collective helplessness in preventing climate change

Variables	Model 3: individual helplessness in tackling climate change				Model 4: City council helplessness in tackling climate change			
	*β*	S.E.	*p* value	Exp(*β*)	*β*	S.E.	*p* value	Exp(*β*)
Gender^a^	−0.401	0.135	0.003	0.669	−0.527	0.149	0.000	0.590
Age^b^			0.000				0.006	
16–24	−0.607	0.291	0.037	0.545	−0.833	0.303	0.006	0.435
25–34	−1.234	0.285	0.000	0.291	−1.048	0.306	0.001	0.351
35–44	−1.064	0.268	0.000	0.345	−0.674	0.267	0.012	0.509
45–54	−0.627	0.290	0.031	0.534	−0.251	0.287	0.383	0.778
55–64	−0.888	0.294	0.002	0.412	−0.451	0.294	0.125	0.637
65–74	−0.349	0.307	0.256	0.706	−0.223	0.298	0.454	0.800
Location^c^	0.479	0.212	0.024	1.614	0.509	0.219	0.020	1.663
HousingTenure^d^	−0.188	0.157	0.230	0.828	−0.281	0.178	0.115	0.755
Voluntary participation in local decision-making groups^e^	−0.235	0.220	0.285	0.791	−0.430	0.256	0.094	0.651
Constant	1.121	0.243	0.000	3.068	−0.028	0.231	0.902	0.972
R^2^ Cox–Snell	0.052				0.050			
R^2^ Nagelkerke	0.070				0.072			
Hosmer and Lemeshow statistic	12.583				8.667			
*p* value	0.127				0.371			
Classification (%)	59.8				70.7			
*N*	943				936			

Reference categories used in the model:

^a^Males

^b^Age group 75+

^c^Outside city center

^d^Owned

^e^Does not participate

### Models 1 and 2: Concern About the Risk of Flooding and Climate Change Affecting Portsmouth

Table 5 shows that the *R*^2^ values are between 0.059 and 0.079 and between 0.131 and 0.178, in models 1 and 2, respectively. The Hosmer and Lemeshow test indicates that the data fit both models well. Finally, the classification rate shows that the percentage of cases predicted correctly by the models is 60.4 for model 1 and 68.2 for model 2.

The results show that the statistically significant determinants of concern with regard to flooding and climate change are similar. The variables which are found to be statistically significant across both models include gender, education, home contents insurance, voluntary participation in local decision-making groups, and individual helplessness. Additionally, in model 1, income is also statistically significant.^9^

With regard to gender, the results of both models indicate that females are more concerned about both flooding and climate change than males. The odds ratio indicates that females are 1.424 times more likely to be concerned about flooding and 1.491 times more likely to be concerned about climate change than males. Education is also statistically significant in both models. Results show that respondents with lower levels of education are less likely to be concerned about both flooding and climate change. The models indicate that respondents with second-level education are 0.492 times less likely to be concerned about flooding and 0.516 times less likely to be concerned about climate change than those who have a third-level education. Additionally, respondents with neither second- nor third-level education are also less likely to be concerned about both flooding and climate change, with odds ratio values of 0.494 and 0.496, respectively.

The possession of home contents insurance is also shown to impact upon reported concerns. Respondents with home contents insurance are 1.82 times more likely to be concerned about flooding and 1.754 times more likely to be concerned about climate change. Additionally, the variable capturing voluntary participation in local decision-making groups indicates that those who participate are 1.646 and 1.890 times more likely to be concerned about flooding and climate change, respectively. The final explanatory variable that is found to be statistically significant in both models is individual helplessness in tackling climate change. Those who report to be individually helpless in tackling climate change are 0.599 times less likely to be concerned about flooding and 0.276 times less likely to be concerned about climate change.

### Models 3 and 4: Perception of Helplessness in Individual and Local Collective Action in Tackling Climate Change

The results of the logistic regressions explaining perceptions of individual and local collective helplessness in tackling climate change are shown in Table 6. The *R*^2^ values are between 0.052 and 0.070 and between 0.050 and 0.072 in models 3 and 4, respectively. The Hosmer and Lemeshow test indicates that the data fit both models well. Finally, the classification rate shows that the percentage of cases predicted correctly in models 3 and 4 are 59.8 and 70.7 %, respectively.

The statistically significant determinants in both models 3 and 4 are the same. Gender, age, and residential location variable are shown to be statistically significant.^10^ The results indicate that females are less likely to perceive both individual and local collective helplessness in tackling climate change. The odds ratios show that females are 0.669 times less likely to perceive themselves to be helpless and 0.590 times less likely to perceive the city council to be helpless in tackling climate change. Moreover, younger respondents are less likely to perceive both individual and local collective helplessness in tackling climate change. Finally, the location variable shows that city center dwellers are 1.614 times more likely to perceive individual helplessness and 1.663 times more likely to perceive local collective helplessness in tackling climate change.

## Discussion

This study has found that it is not always the same individuals who are concerned about both climate change and flooding. The finding that not all residents who reported concern about climate change are concerned about flooding, and vice versa, points, perhaps, to a lack of knowledge on the relationship between the two environmental threats. This is the case even in a city at substantial risk of, and experience of, flooding. This is consistent with previous research which found that the public lack an understanding of climate change and its effects (see for example Kempton et al. 1996; Reynolds et al. 2010).

General knowledge is shown to play a role in explaining concern about both flooding and climate change. The regression results show that education increases the probability of reporting concern, which is consistent with previous research (Tjernstrom and Tietenberg 2008; Wu and Cutter 2011; Milfont 2012). Moreover, the strength of the effect of education is found to be similar in explaining concern about both flooding and climate change. Education levels are often taken as an indicator of general knowledge, and the findings indicate that this increased knowledge does impact upon both concerns.

Gender is also statistically significant in explaining concern about both flooding and climate change. Females are more likely to express concern than males. This finding is consistent with the risk perception literature which found that women are, in general, more concerned by risk than males (O’Connor et al. 1999; Kellstedt et al. 2008). Moreover, gender contributes to explaining differences regarding concern about both threats. The odds ratio on the gender variable indicates that gender is somewhat stronger in explaining concern about climate change than flooding.

Voluntary participation in decision-making groups is also statistically significant in impacting upon concern about both flooding and climate change. This prosocial, less individualistic, or altruistic behavior is associated with greater concern. The strength of the effect of this variable is found to be stronger in explaining concern about climate change, the global threat. The possession of home contents insurance indicates the willingness of individuals to take individual action against typically insurable risks. The results show that these individuals are more likely to be concerned about both flooding and climate change. However, the odds ratios show that the effect is stronger in explaining concern about flooding, the local threat.

The final statistically significant variable in explaining concern about both flooding and climate change is perceptions of individual helplessness in tackling climate change. Respondents who reported helplessness are less likely to be concerned about both flooding and climate change. Indeed, helplessness impacts differently on reported concerns. Those who perceived themselves helpless are far less likely to be concerned about climate change than they are about flooding. The results suggest that respondents who do not think they can do anything about climate change do not concern themselves with either climate change or flooding. Moreover, respondents are less likely to concern themselves with climate change, the less salient threat. Although, the use of the term “tackling climate change” in the survey question does not allow a distinction to be made between mitigation and adaption efforts, it does however indicate differences in public perceptions of helplessness with regard to doing something about climate change. Lorenzoni et al. (2007) found that those who perceive themselves as helpless do not take action; in fact, it is shown here that they are not even likely to be concerned. A more nuanced understanding of perceptions of helplessness is, as such, useful.

In examining perceptions of helplessness in tackling climate change, respondents reported helplessness at the individual and local collective levels differently. While 51.5 % of residents perceived themselves helpless in tackling climate change, only 28.5 % perceived helplessness at the local collective action level. This proportion of reported helplessness, at both levels, is perhaps low given the recognized difficulties in individualistic and collective action remedies to the climate change problem (Libecap 2014). As such, Portsmouth residents are less fatalistic with regard to their helplessness in the face of global climate change and the threat their city faces. It may be the case that respondents do not perceive the collective action problem to be constraining on their individual ability, and less so on the ability of local collective action, to tackle global climate change. This is consistent with some of the experiments in the economics literature which suggested that individuals are more inclined to act collectively than economic theory predicts (see Ledyard 1995 for a review). As such, respondents may be more altruistic in their behavior than the individualistic approach to public goods suggests, and their low reporting of perceptions of helplessness may be better explained by allowing for an altruistic approach, such as in Andreoni (1989, 1990). On the other hand, the regression models do not find the civic activism variable to be statistically significant in explaining helplessness.

Moreover, the number of respondents reporting helplessness at the local collective level is substantially lower than that at the individual level. This is the case despite the fact that local collective action is limited in contributing to a global public good. This result indicates that the mechanisms necessary to ensure provision of a global public good may not be well understood, or, alternatively, this may be due to respondents recognizing that in tackling climate change, local collective action can be effective in adapting to climate change, for example through flood defenses. The conclusion that can be drawn here is limited by the design of the survey instrument on helplessness.

In explaining the determinants of helplessness, the results show that females are less likely to perceive helplessness in tackling climate change at the individual and local collective levels. The impact of gender is stronger at the local collective level. Additionally, younger respondents are found to be less likely to perceive individual and local collective helplessness in tackling climate change. Considering the global public good context of tackling climate change, these results indicate that females and younger respondents may be less conscious of both their individual and the local city council’s helplessness. An alternative explanation is that these respondents may be less fatalistic in their views regarding climate change than males or older respondents.

## Concluding Remarks

This study reports on attitudinal data from the residents of Portsmouth on their concerns and perceptions of helplessness in the face of climate change and flooding. Similarly, high proportions of residents have been found to be concerned about both climate change and flooding in this low-lying island city at risk of inundation and with recent experience of flooding. With regard to perceptions of helplessness in tackling climate change, substantially more residents perceived themselves to be helpless than viewing the local council to be helpless.

The results show that those who are more likely to express concern about both flooding and climate change are females, those having higher levels of education, those holding home contents insurance as well as those who volunteer. Moreover, respondents who expressed perceptions of individual helplessness in tackling climate change are less likely to be concerned with climate change or flooding. In explaining helplessness, females, younger respondents, and those residing outside the city center are less likely to report helplessness in tackling climate change both as individuals and through local collective action.

As other cities across the globe face climate change challenges, these findings, based upon attitudes from an alert urban population, are informative to policy design. Individuals are found to view local collective action to be more effective in tackling climate change than individualistic action. Rather than a fatalistic outcome, which arises from an individualistic approach to the collective action problem, the results seem to show some broad alignment with the environmentalist adage—“thinking globally, acting locally”—but particularly where the ‘local’ pertains to local collective action channeled through local authorities. Public messages and policy communication on the environmental threats discussed could potentially harness these findings to more effectively build support. This approach seems likely, in this context, to be more generally effective than focusing attention and resources on trying to elicit more atomistic, individual behavioral changes and support.

The role of education points to the usefulness of public policy actions to increase understanding of both environmental threats and their interrelationship. This could be particularly useful as it is shown that education is not related to perceptions of helplessness in tackling climate change, despite its role in determining concerns about it. This is a positive finding from a policy perspective as this indicates that information (education) leads to greater concern regarding flooding and climate change but has no impact on perceptions of helplessness. This could be most effectively achieved by targeting public messages and public communications to females, younger people, and those already participating in voluntary activities.

The findings presented here are limited to only one instrument to measure helplessness and concern. Additionally, no distinction is made in reported helplessness with regard to efforts to mitigate or efforts to prevent climate change. It would be useful for future research to develop our understanding of perceptions of helplessness and concerns with regard to environmental threats in different settings, and also to focus on disentangling perceptions of helplessness with respect to mitigation and adaption efforts to tackle climate change.

## Footnotes

1 Psychological distance increases along key dimensions including spatial or geographical distance, temporal distance, social distance between perceiver and target, and uncertainty. Spence et al. (2012) provide a useful overview of construal level theory and its application to risk perceptions associated with climate change.

2 See Hair et al. (2007, p. 178) on stratified sampling methodology.

3 This study is limited to only one measure for each dependent variable.

4 The results of this multinomial model are not presented as they added little to the results from models 1 and 2. The results did show that the findings from models 1 and 2 are robust.

5 For each of the dependent variables outlined in Table 1, the four categories have been collapsed to two. Categories 1 and 2 are collapsed to represent an affirmative answer, while categories 3 and 4 are collapsed to represent a negative response.

6 A drawback of collapsing variables is that there is the risk of losing useful information. Hence, for robustness purposes, multinomial regression models were also estimated. This approach is also not without its drawbacks, as these models “…become inefficient and become increasingly difficult to interpret as the number of categories in the dependent variable rises because of the large number of parameters that are estimated” (Lewis-Beck et al. 2003, p. 941). The results from the multinomial regressions are qualitatively similar to the results obtained using binary logistic regression models. Hence, for brevity and ease of exposition, the results from the binary logistic models are presented here. All regression results are available upon request.

7 Rubin (1977) proposed using the method of multiple imputations to calculate missing income observations. According to Rubin (1977) multiple imputation “…produces a subjective probability interval for the statistic that would have been calculated if all non-respondents had responded. Background information which is recorded for both respondents and non-respondents plays an important role in sharpening the subjective interval. The general idea can be applied to any problem with non-respondents or missing data” (p. 538). Tabachnick and Fidell (2007) regard this method as the most reputable method to deal with missing data.

8 No single variable across the regressions considered has a variance inflation factor (VIF) higher than the common set threshold of 10, thereby satisfying the rule of 10 (O’Brien 2007).

9 With the exception of the voluntary participation variable in model 1 and the income variable, the results are robust to Bonferroni corrected *p* values for $0.05/n$, where *n* is the number of hypotheses tested.

10 With the exception of the location variable, the results are robust to Bonferroni corrected *p* values for $0.05/n$,where *n* is the number of hypotheses tested.
